# Wear-Resistant Smart Textiles Using Nylon-11 Triboelectric Yarns

**DOI:** 10.1021/acsami.3c14156

**Published:** 2023-11-20

**Authors:** Piotr K. Szewczyk, Tommaso Busolo, Sohini Kar-Narayan, Urszula Stachewicz

**Affiliations:** † Faculty of Metals Engineering and Industrial Computer Science, AGH University of Krakow, Krakow 30-059, Poland; ‡ Department of Materials Science & Metallurgy, University of Cambridge, Cambridge CB3 0FS, United Kingdom

**Keywords:** triboelectric devices, smart textiles, energy harvesting, electrospinning, PA11

## Abstract

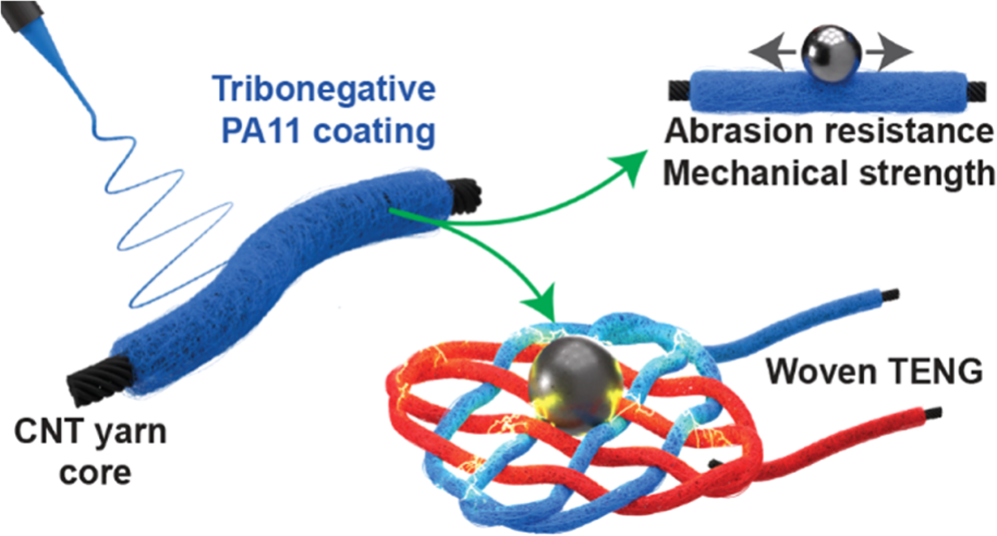

The ever-increasing demand for self-powered systems such as glucose biosensors and mixed reality devices has sparked significant interest in triboelectric generators, which hold large potential as renewable energy solutions. Our study explores new methods for integrating energy-harvesting capabilities into smart textiles by developing strong and efficient yarns that can convert mechanical energy into electrical energy through a triboelectric effect. Specifically, we focused on Nylon-11 (PA11), a material known for its crystalline structure well-suited for generating a powerful triboelectric response. To achieve this, we created triboelectric yarns by electrospinning PA11 fibers onto conductive carbon yarns, enabling energy-harvesting applications. Extensive testing demonstrated that these yarns possess exceptional durability, surpassing real-life usage requirements while experiencing minimal degradation. Additionally, we developed a prototype haptic device by interweaving tribopositive PA11 and tribonegative poly(vinylidene fluoride) (PVDF) triboelectric yarns. Our research has successfully yielded durable and efficient yarns with strong energy-harvesting capabilities, opening up possibilities for integrating smart textiles into practical scenarios. These technologies are promising steps to achieve greener and more reliable self-powered systems.

## Introduction

**Figure 1:**
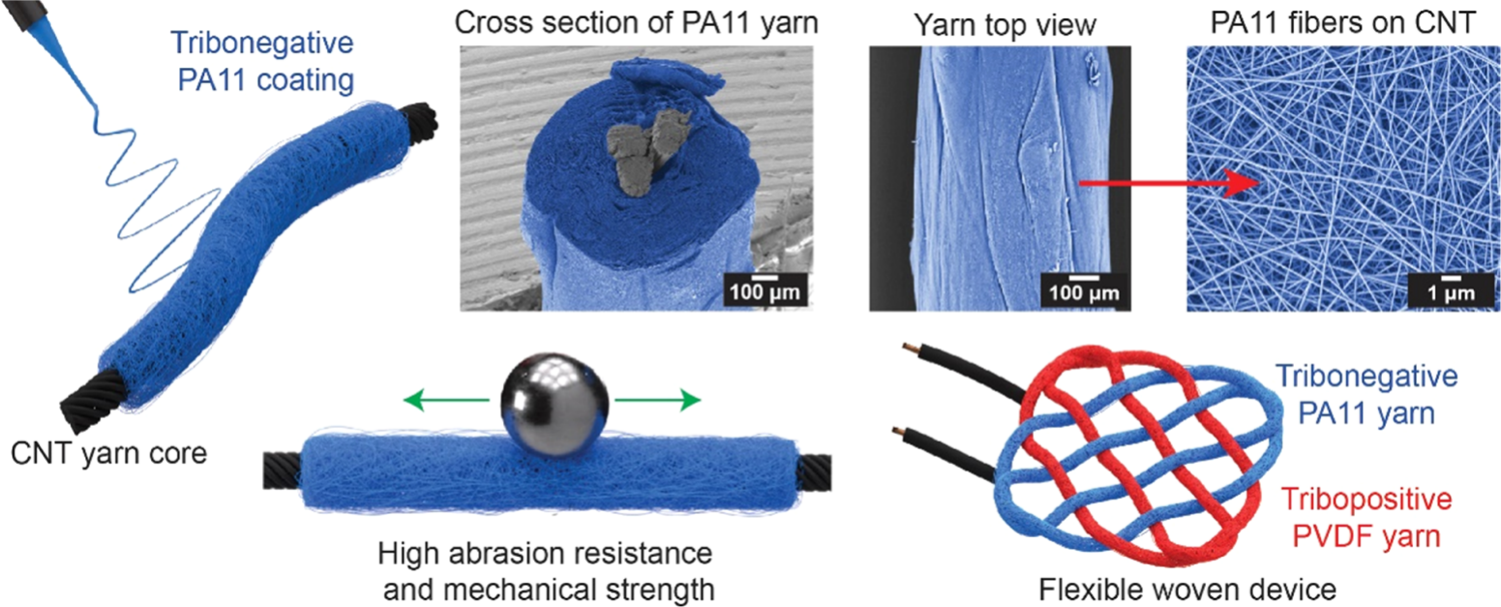
Conceptual schematic of the study on tribopositive yarn based on electrospun PA11 fiber-based coating on a conductive carbon nanotube (CNT) core wire for constructing high wear and water resistance energy-harvesting devices. SEM images have faux colors for clarity.

As our use of portable and wearable electronics grows, we must rethink current power generation and energy management methods. Among the various technologies designed to harvest energy, triboelectric nanogenerators (TENGs) stand out due to their unique ability to turn surrounding mechanical energy into electricity. It is essential when considering the future of smart textiles: fabrics integrated with electronic components. A TENG functions based on the principle of contact and separation between two dissimilar materials, usually with contrasting electron affinities. When these materials come into contact, they generate electric charges through the transfer of electrons between them. Upon separation, the charge separation leads to a potential difference that generates an electrical current, thereby producing power.^1^ This cyclic contact and separation process continually generates electric charges and, when connected to an external circuit, facilitates the harvesting of electrical energy. Incorporating TENGs into these materials could reduce our reliance on bulky batteries and lessen the need for frequent charging.^1^ Beyond energy generation, TENGs also have the potential to function as self-powered sensors, opening up new possibilities for advanced, interactive electronic devices.^2^ Triboelectric devices have been mainly fabricated on flexible polymer substrates, but they can also be made into yarns by coating a conductive core with a triboelectric material.^3,4^ These yarns can be woven or knitted together to create functional triboelectric textiles.^4^ Consequently, it is important that TENGs designed for smart textiles possess the resilience to endure textile manufacturing processes, offer comfort during wear, and remain washable for maintenance-free and seamless integration.^5^ Use of carbon nanotube (CNT) yarns offers exceptional specific strength and conductivity, which are vital for ensuring the flexibility, robustness, and longevity of triboelectric textiles under real-world conditions.^6^ Moreover, CNT yarns provide a unique advantage in facilitating seamless interconnectivity between yarns and other textile components, eliminating the need for conventional rigid joining methods like soldering or conductive epoxy, which are necessary for solutions with a metallic core.^3^ As for the shell material, combining tribopositive and tribonegative materials into a textile is ideal for high-energy-harvesting performance.^4^ Several tribonegative yarns have been demonstrated, mainly based on poly(vinylidene fluoride) (PVDF) and poly(dimethylsiloxane) (PDMS).^6−8^ However, only a handful of tribopositive yarns have been reported due to the limited number of tribopositive synthetic materials (*e.g.*, nylons, polypropylene) and their challenging fabrication process.^9,10^ To facilitate the real-world application of functional triboelectric textiles, it is essential to develop durable, high-power tribopositive yarns, as illustrated in Figure 1.

Nylon-11 (PA11) is a promising polymer for energy harvesting due to its mechanical resilience and triboelectric properties.^11^ The mechanical properties of PA11 are crucial for ensuring the durability and performance of triboelectric textiles, allowing them to withstand the demands of daily use while efficiently harnessing energy. However, solution processing of PA11 has been a major hurdle for film and fiber-based devices.^12^ There are several phases of PA11, namely, α, β, γ, and δ′, but only highly aligned α and δ′ have high polarization needed for high triboelectric output. Complex methods using extremely rapid evaporation rates or nanoconfined templates are required to produce aligned α and δ′ phases.^13,14^ Electrospinning offers a promising fabrication route because of its unique combination of rapid evaporation and mechanical stretching. The nonwoven materials produced this way consist of a large network of interconnected fibers, providing a highly developed surface essential for achieving high charge density, a crucial factor for TENGs.^15^ Anwar et al. demonstrated that α and δ′ phase PA11 fibers can be fabricated by dissolving the PA11 in a trifluoroacetic acid(TFA)/acetone solution and adjuring the polymer concentration.^16^ Electrospinning is a promising method to fabricate triboelectric yarns because of its scalability, broad range of available materials, and potential to enhance a material’s triboelectric and mechanical performance in a one-step process.^17−19^ Triboelectric yarns can be produced by depositing polymer fibers directly on a conductive yarn using a high electric field and tribonegative PVDF-based yarn with high-power output and superior resistance to fatigue, and abrasion was already reported in a previous study.^6,7,20^

In this work, we developed a novel highly tribopositive yarn based on an electrospun PA11-coating on a conductive carbon nanotube (CNT) core. The resulting yarn displayed remarkable energy-harvesting performance and high wear and water resistance. Finally, PA11 tribopositive yarns were woven with PVDF tribonegative yarns to create a triboelectric textile that demonstrated its real-life applicability in energy harvesting and sensing, such as in a human–computer interface. The schematic showcasing the core idea and novelty of the study is presented in Figure 1.

## Results and Discussion

### Morphology, Mechanical Properties, and Chemical Composition of PA11 Fibers

The fabrication and real-life use of triboelectric yarns necessitates materials with high toughness and stretchability beyond 20% strain.^1^ Fibrous meshes, such as electrospun PA6, have shown higher toughness and crack resistance than film counterparts, emphasizing the advantage of fibrous meshes for stable triboelectric layers.^21^ Polyamides are known for high mechanical stability and are widely studied in fibrous form.^22,23^ It is vital to achieve highly crystalline materials to optimize the energy-harvesting properties. PA11 can exist in several crystalline phases, including triclinic α-phase, monoclinic β-phase, and three hexagonal or pseudohexagonal forms (γ-, δ-, and δ′- phases). Fabricating PA11 fibers with polar phases, such as aligned γ and δ’, is essential to optimize their energy-harvesting properties.^24,25^ In electrospinning, relative humidity (RH) conditions can significantly affect the rate of solvent evaporation.^26^ Higher RH levels slow solvent evaporation from the polymer solution jet, leading to delayed solidification of polymer fibers. This delay causes structural variations in the fibers, impacting their mechanical properties, crystallinity, and other characteristics, ultimately affecting the material’s energy-harvesting capabilities.^26^ RH influence on the rate and nature of PA6 formation, fiber structure, and the material’s mechanical properties has been previously reported.^26−28^ Thus, we conducted experiments under RH conditions of 30, 50, and 70% to investigate their influence on the properties of self-standing electrospun PA11. The prepared fiber samples were labeled F30, F50, and F70 to reflect the RH at which they were produced.

**Figure 2:**
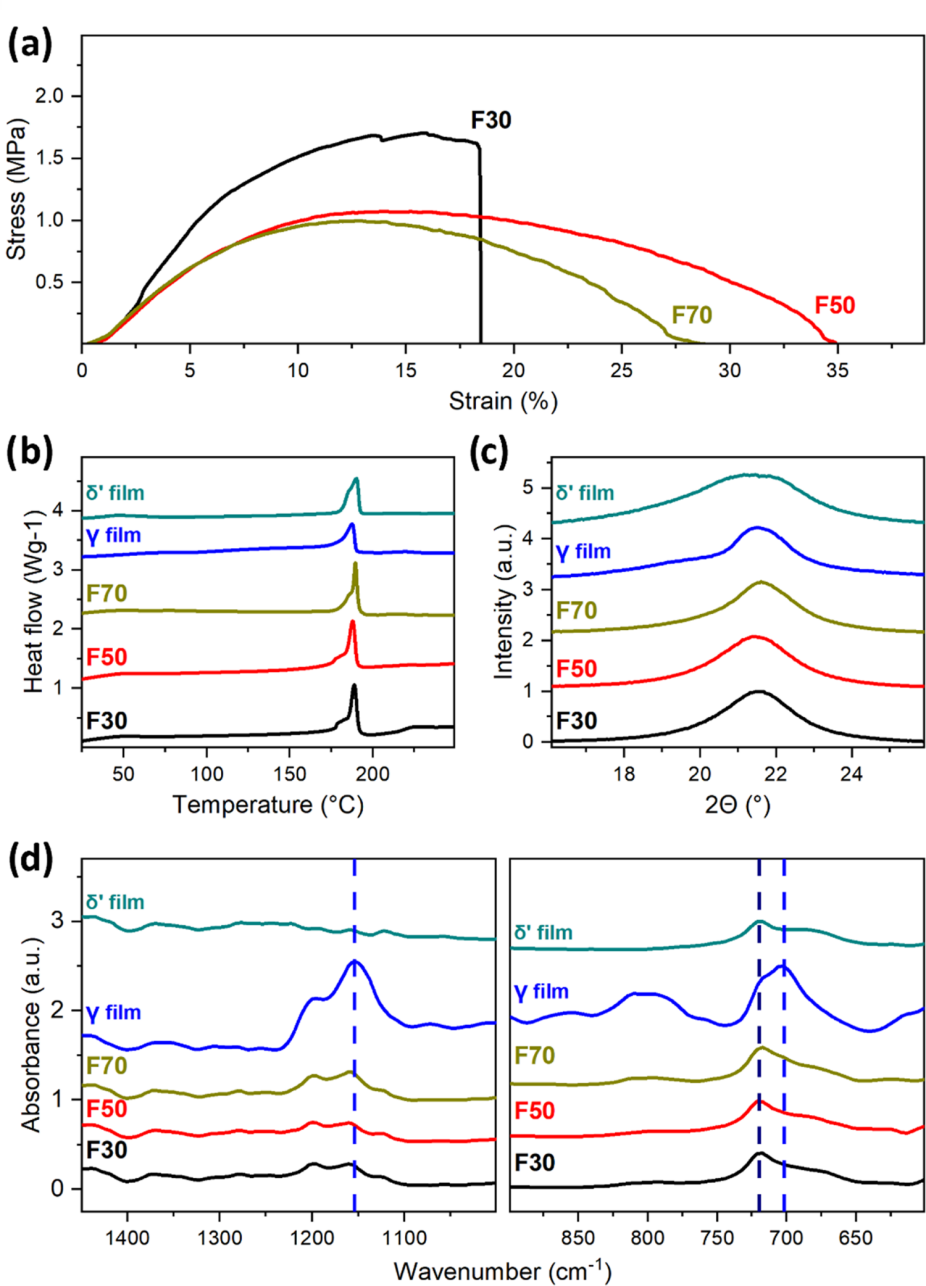
(a) Exemplary stress–strain plot of tensile tested self-standing PA11 fiber networks, (b) DSC characterization of PA11 fibers, γ and δ′ phase films (first heating), (c) XRD characterization of PA11 fibers, γ and δ′ phase films, (d) structural characterization of PA11 fibers and γ and δ′ phases using FTIR with magnified spectra of the 550–900 cm^–1^ and 1400–1000 cm^–1^ regions. The dashed red line is a visual indicator of the CO–NH skeletal motion peak (P1) at 1156 cm^–1^. The dashed blue line is a visual guideline for the δ′ phase amide V band (686 cm^–1^). The dashed black line serves as a visual guideline for the γ phase amide V band (709 cm^–1^).

A process that creates uniform, defect-free fibers is essential for a robust mesh. Thus, scanning electron microscopy (SEM) imaging was used to assess the PA11 fibers’ shape and diameters; see Figure S1. The electrospun mats exhibited a typical fibrous morphology under all tested RH conditions. All fibers had a smooth surface without visible beads. The diameters for all samples ranged from 100 to 145 nm, with narrow distributions indicating stable electrospinning. The thickness of the mats showed minimal variation. Specifically, for F30, F50, and F70, the thickness measurements were 34.3 ± 3.7, 37.0 ± 1.7, and 35.5 ± 2.0 μm, respectively. Similarly, the porosity remained consistent across different humidity levels. The values were 40.3 ± 2.8% for F30, 36.7 ± 0.5% for F50, and 37.1 ± 2.1% for F70, indicating a negligible difference. Thus, varying RH did not affect the morphology, porosity, or fiber diameters in a significant way. Our results align with previous reports showing excellent reproducibility of electrospun PA11 fibers.^24^ The tensile test results are presented in Figure 2a and Table S1. Fibers fabricated at 30% RH demonstrated the lowest strain at failure, 21.2 ± 2.5%, with a maximum tensile stress of 1.79 ± 0.32 MPa. The toughness of fibers produced under these conditions was 28.3 ± 6.7 MPa. An increase in strain at failure to 28.2 ± 1.9% was observed for the fibers prepared at 70% RH, underlining the influence of the humidity-dependent formation process on the material’s mechanical characteristics.^29^ However, the ultimate tensile stress corresponding to this condition was the lowest among the three, registering at 0.97 ± 0.03 MPa. The toughness also decreased to 18.0 ± 0.1 MPa at this RH. PA11 fibers produced at 50% RH provided an ideal blend of mechanical properties. They exhibited the strain at failure at 35.5 ± 1.0%, double that observed for PA11 fibers produced under 30% RH. In addition, maximum tensile stress was measured at 1.02 ± 0.14 MPa for this condition, and toughness declined slightly to 25.8 ± 0.9 MPa compared to PA11 fibers produced under 30% RH. These results indicate that 50% RH could be an optimal environment, achieving the best strain at failure without compromising toughness. Compared to PA6 tested under the same conditions, our PA11 fibers demonstrated superior mechanical properties, especially for the strain at maximum stress and toughness, making them a more suitable option for TENG applications.^30^

To understand the changes in mechanical properties, we investigated the thermal properties, and crystallinity of PA11 fibers using differential scanning calorimetry (DSC) (Figure 2b). Melting points ranged from 187.5 °C (γ phase) to 190 °C (δ′ phase) for all samples, suggesting a mixed crystal structure. F30 and F50 fibers displayed an additional peak at 181 °C, likely corresponding to the second δ′ phase melting peak observed at 185 °C in the reference film. This implies a higher fraction of δ′ phase in these fibers compared to the F70 sample. The first melting peaks were utilized to determine fiber sample crystallinity. F30 and F50 samples exhibited a higher crystallinity of 41 ± 1 and 43 ± 4%, in contrast to 27 ± 0.3% found in the F70 sample, while γ and δ′ phase films showed values of 26 ± 2% and 17 ± 2%, respectively. The enhanced crystallinity of electrospun PA11 arises from high elongation strain, shear forces, and a strong electric field during the electrospinning process. This results in a highly organized fiber structure composed of aligned crystallites with amorphous regions filling the gaps.^31^ The crystal structure of the fibers was further probed using X-ray diffractometry (XRD) (Figure 2c). The F30 and F50 fibers displayed a single broad peak at approximately 21.4°, a combination of the (100) reflection of γ phase and the (100) reflection from the δ′ phase. In contrast, the F70 fiber had a slightly shifted peak center (21.6°), suggesting a lower δ′ phase volume fraction and a shift toward the γ phase composition, which aligns with DSC data. Moreover, the PA11 fiber samples exhibited a more organized pseudohexagonal structure than films, as evidenced by a smaller full width at half-maximum of the δ′-phase line, 2.44–2.59 and 3.8 for fibers and δ′ film, respectively. Similar composition of PA11 electrospun from hexafluoroisopropanol (HFIP) has been reported, suggesting electrospinning leads to higher amounts of beneficial δ′-phase due to high electric field and mechanical stretching.^32^ Crystal structure investigation was continued by using Fourier transform infrared spectroscopy (FTIR). Figure S2 shows the spectra of PA11 fibers compared to those of γ and δ′ phase films. PA11 has characteristic primary and secondary amide bands that appear in the 1500–1800 cm^–1^ region and are associated with hydrogen-bonded or free amide groups. In addition, the vibrations at 3300 and 1639 cm^–1^ correspond to the N–H and C=O stretching modes. The 2920 and 2850 cm^–1^ vibration bands originate from the antisymmetric and symmetric CH_2_ stretch.^25^Figure 2d provides further insight into the RH effects on PA11 fibers. We can observe that increasing humidity affected both peak position and intensity, especially in regions around 700 and 1200 cm^–1^ wavenumbers. All of the samples had a broad peak around 700 cm^–1^, composed of an intense peak at 721 cm^–1^ (CH_2_ rocking) next to a lower intensity peak for the amide V band, found at 709 and 686 cm^–1^ for the γ and δ′ phases, respectively. In the F30 and F50 fibers, the amide V band was located at 686 cm^–1^, while in the F70 sample, this band shifted toward 709 cm^–1^. This indicates that the F30 and F50 samples had a higher fraction of the δ′ phase compared to the F70 sample. In the CH_2_ progression bands region (1000–1400 cm^–1^), the CO–NH skeletal motion peak (P1) was found at 1153 and 1161 cm^–1^ for the γ and δ′ phase films, respectively. The P1 peak was located at 1161 cm^–1^ for F30 and F50 samples but shifted to 1157 cm^–1^ in the F70 sample. This further shows that the F30 and F50 samples have a higher δ′ phase content compared with the F70 counterpart. Based on the shifts in the characteristic amide V bands (709 and 686 cm^–1^ for γ and δ′ phases, respectively) as well as the shift toward the γ phase in the 1100–1200 cm^–1^ region, the data confirms the presence of both γ and δ′ phases. These results suggest that the PA11 fibers are highly crystalline and contain a mixture of both γ- and polar δ′ phases, which aligns with XRD and DSC results. Furthermore, the presence of a metastable δ′ phase indicates extremely high evaporation of solvents during electrospinning.^25^

In summary, all produced fibers met the above 20% strain at failure criteria, with the 50% RH condition found to be optimal for making highly mechanically stable fibers. This disparity in mechanical properties was driven by changes in the crystalline structure of the obtained fibers.^33^ The DSC analysis revealed that all samples had a mixed crystal structure composed of γ and δ′ phases. Notably, F30 and F50 exhibited an additional δ′ phase melting peak at 181 °C, indicating a higher δ′ phase fraction compared to F70. In the XRD analysis, the F30 and F50 fibers displayed a broad peak at 21.4°, representing both γ and δ′ phase reflections. Confirming DSC results, the F70 sample had a peak centered at 21.6°, suggesting a shift toward a greater amount of γ phase in the composition. FTIR spectra further confirmed our findings and showed notable differences in the amide V band. F30 and F50 samples exhibited this band at 686 cm^–1^, while F70 had it at 709 cm^–1^, indicating higher δ′ phase content in the former. Because formic acid and water readily form hydrogen bonds, it is probable that different humidity levels influenced the evaporation rate of formic acid, subsequently impacting the crystalline structure of the electrospun material.^27^ Our results are analogous to research carried out on PA6 by Giller et al.^27^ in which fast and slow solvent evaporation resulted in distinct changes in the phase composition of the prepared fibers. Thus, the influence of RH has been confirmed to affect more than one type of polyamide. The presented research outcomes underscore that careful control of preparation conditions can effectively tailor the mechanical stability and phase structure of PA11, demonstrating the advantages of electrospinning.^34^

### Energy Harvesting and Surface Potential of PA11 Fibers

**Figure 3:**
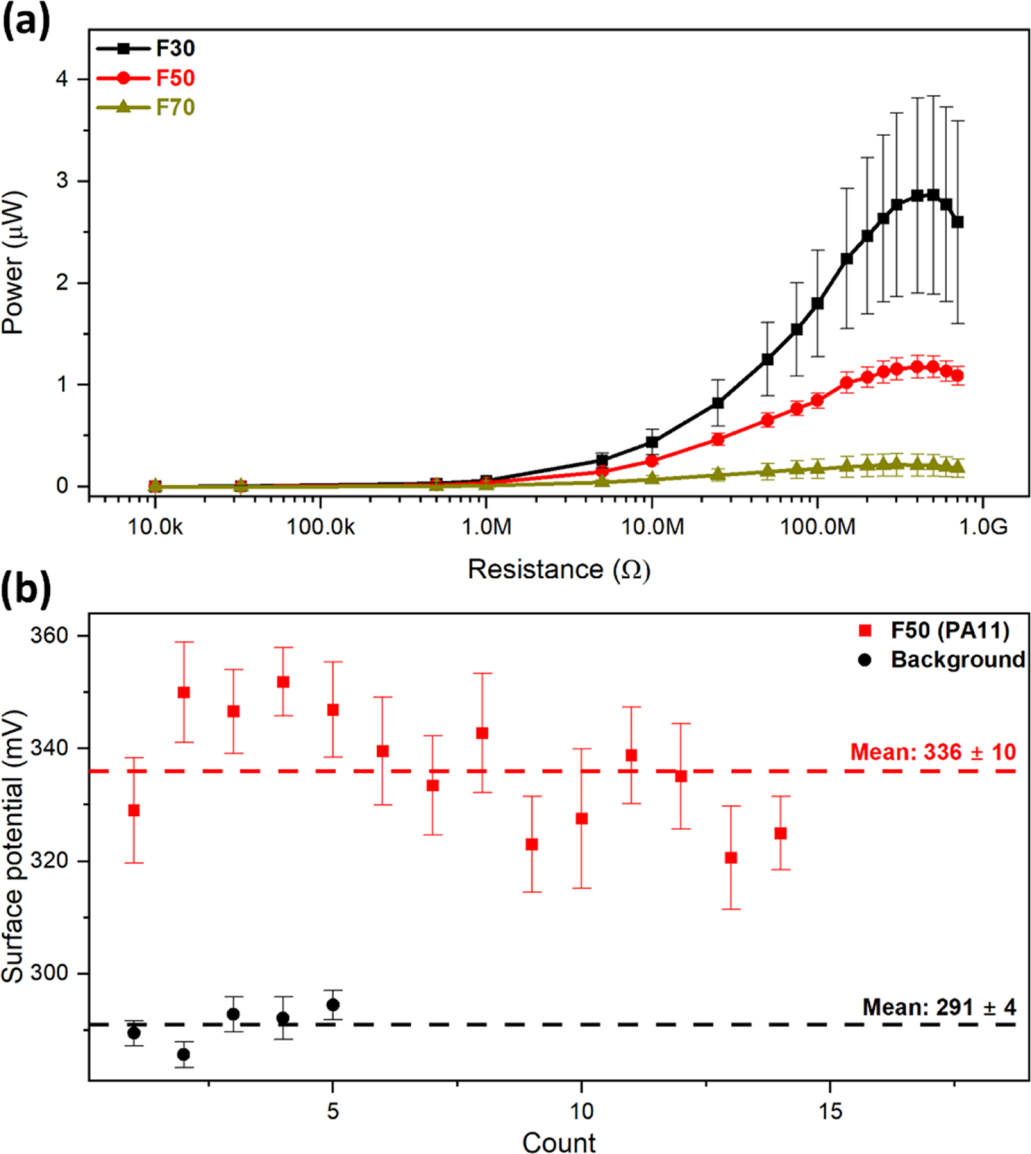
(a) RMS power output measured across several load resistances of PA11 fibers produced with different RH and (b) surface potential plot of PA11 fibers compared to ITO glass background.

To understand RH’s influence on the energy-harvesting capability of triboelectric generators, we assembled several devices and tested their power output; see Figure 3a. We recorded the power output for the F30 sample with 2.87 and 3.34 μW across a 500 MΩ resistor, respectively. In the F50 device, as it produced a maximum power output of 1.18 and 0.99 μW across a 500 MΩ load resistance. The power output drastically dropped for F70, having a maximum power output of 0.21 μW across a 300 MΩ load resistance. The results showed that RH strongly influenced the power output of the devices. Lower RH led to an increase in the power output of the triboelectric devices. Such a result correlated with the increase in crystallinity and amount of the polar δ′ phase as characterized by DSC, XRD, and FTIR. While all samples had similar thickness, fiber diameters, and porosities, ruling out variations in the contact area as a factor, we observed that the F30 sample delivered a higher power output than the F50 despite having comparable degrees of crystallinity. This disparity can be attributed to variations in the ratio of the γ and δ′ phases in the fibers, as suggested by DSC and XRD profile fitting results. The significant variation observed in the F30 sample suggests instability and the potential for degradation over extended periods, raising doubts about its suitability for real-world applications. This instability is reflected in the tensile testing results, where the F30 sample performed the worst, barely exceeding 20% elongation at the breaking point. Therefore, lower mechanical performance of the F30 sample leads to rapid degradation during triboelectric testing. In TENG, stability and flexibility are essential. Thus, we decided to focus our further characterization efforts exclusively on the F50 sample, as it demonstrated the best combination of mechanical properties and triboelectric performance.

Kelvin probe force microscopy (KPFM) measurements were carried out to test the spread of the surface potential between individual fibers, which might indicate differences in the structure and potential instabilities. Exemplary atomic force microscope (AFM) topography scan and the accompanying surface potential map are shown in Figure S3. All tested fibers exhibited similar surface potentials with an average of 336 ± 10 mV (Figure 3b). Such a small standard deviation indicates high homogeneity of the surface potential of all prepared fibers. Thus, we conclude that all produced fibers possess similar properties and chemical composition. A similar value of approximately 510 mV was already reported for δ′ phase-rich PA11 nanowire films.^11^ A high positive surface potential makes PA11 fibers an excellent candidate for TENG with a negative pair such as PVDF, which has reported negative voltage potential (as measured with KPFM) in the range of around −100 to −400 mV depending on the processing conditions.^35,36^

### Yarn Fabrication and Characterization

To demonstrate the PA11 potential for real-world applications, a triboelectric yarn was fabricated by electrospinning PA11 fibers directly onto a carbon nanotube (CNT) yarn, creating a core–shell structure.^6^ Based on the results from previous sections, we have chosen fibers electrospun at 50% RH (F50) as the coating as they displayed the optimum balance between mechanical properties and triboelectric performance. In this process, the CNT yarn was placed perpendicular to the electrospinning nozzle, which was scanned along the length of the CNT yarn to deposit the PA11 fibers. The yarn schematic with SEM images of the end product is shown in Figure 1. The yarns had an average diameter of 713 ± 51 μm and were coated with PA11 to a thickness of approximately 440 μm. The coating surface exhibited relative uniformity, with occasional creases along the length of the yarn. At the individual fiber level, the surface of the PA11 fibers was smooth, and their average diameter was 124 ± 21 nm (Figure S4). Notably, the formation of PA11 fibers was unaffected by modifications to the electrospinning setup or the use of a CNT yarn as a collector compared to standard electrospinning procedures used earlier in this study.

### Energy-Harvesting Performance and Stability of Yarns

The energy-harvesting performance combined with the mechanical stability of the tribopositive PA11 triboelectric yarn was characterized by tapping the yarn against a poly(tetrafluoroethylene) (PTFE) film as the tribonegative material. The working principles of PA11 triboelectric yarns and a difference in working principle to self-standing meshes are shown in Figure S5. The working principle of the PA11 yarn was nearly identical to that of the previously reported PVDF yarn.^6^ As the yarn contacts the PTFE film, charges are transferred from the PA11 fibers to the PTFE film. The PA11 fibers become positively charged, and the PTFE film accumulates negative surface charges. When the yarn and film are separated, a potential difference develops and the charges on the surface of each material induce an electron flow in the respective electrode.

**Figure 4:**
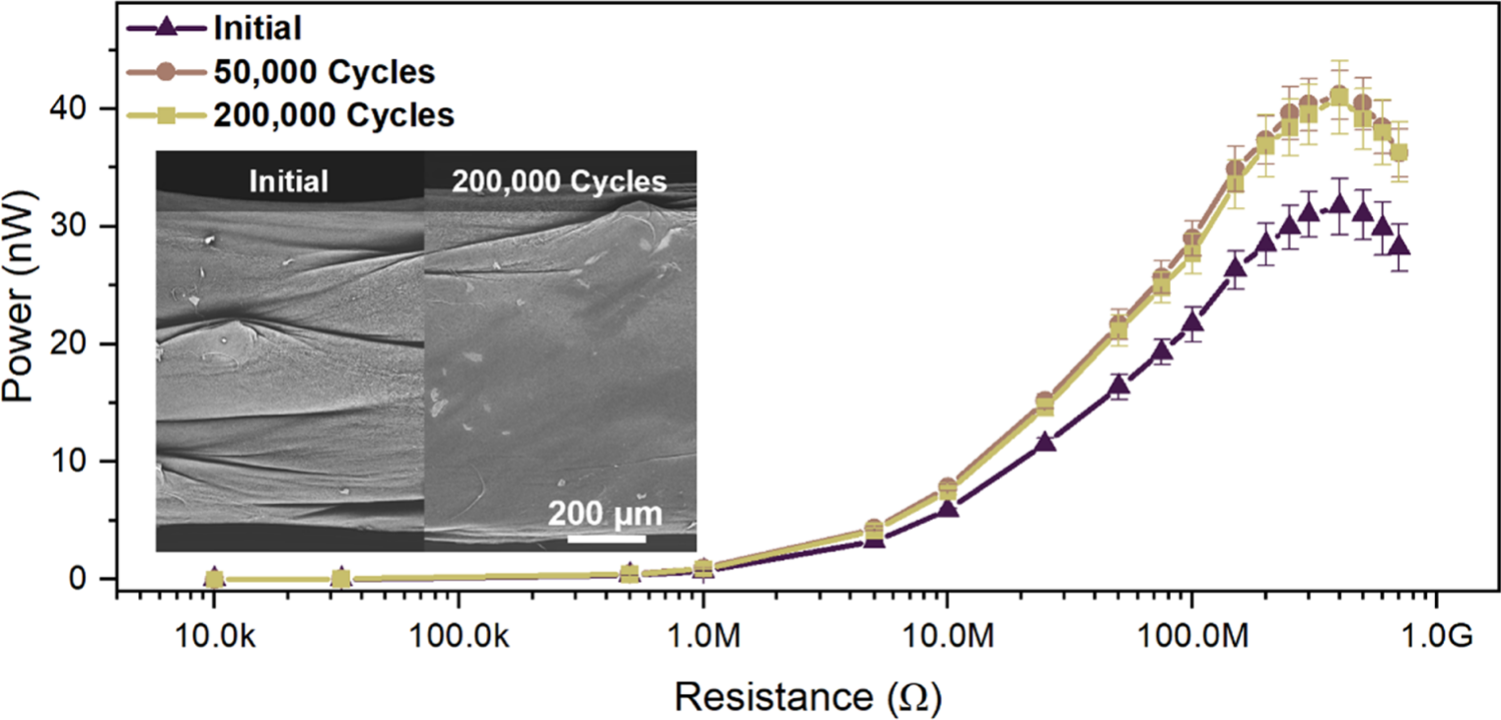
Energy-harvesting performance and durability of the PA11 triboelectric yarn. (a) RMS power output of the triboelectric yarn across different resistors during fatigue testing with SEM images of the same triboelectric yarn as fabricated and after 200,000 tapping cycles.

The results in Figure 4 showed that the yarn initial power output was 31.7 nW and it increased to 41.2 and 40.9 nW after 50,000 and 200,000 cycles. After the test, the yarn surface was inspected using SEM. The images showed that the yarn diameter increased from 757 to 837 μm after 200,000 tapping cycles, but no coating failure was observed. Furthermore, the PA11 fibers deformed into a film-like structure, lowering the fiber network porosity from 34.1 ± 2.6 to 20.7 ± 3.0%. Irregularities on the yarn coating such as creases were flattened due to repeated mechanical deformation. The increase in total contact area with fatigue cycles (10% larger yarn diameter and 40% reduction in fiber network porosity) is linked to a 30% increase in the yarn power output. Similar fatigue behavior was reported for electrospun triboelectric yarns.^20^ It is challenging to directly compare the power density of the triboelectric yarn with previously reported yarns because of the lack of standardized testing parameters (*e.g.*, counter material, force, and contact area) and the challenges in quantifying the yarn contact area (due to fiber network porosity). However, a qualitative comparison was performed by estimating the contact area (7.3 mm^2^), and the results showed that after 200,000 fatigue cycles, the peak power density obtained from the triboelectric PA11 yarn was 121 mW m^–2^ across a 500 MΩ resistor. The results showed that the PA11 triboelectric yarn had a relatively lower power output compared to previously reported electrospun triboelectric yarns when subjected to similar mechanical excitation.^6^ However, this was expected, considering that the PA11 yarn had a lower contact area due to a smaller yarn diameter. Nevertheless, the triboelectric power output of the tribopositive PA11 yarn was comparable to previously reported tribonegative yarns.^37^

**Figure 5:**
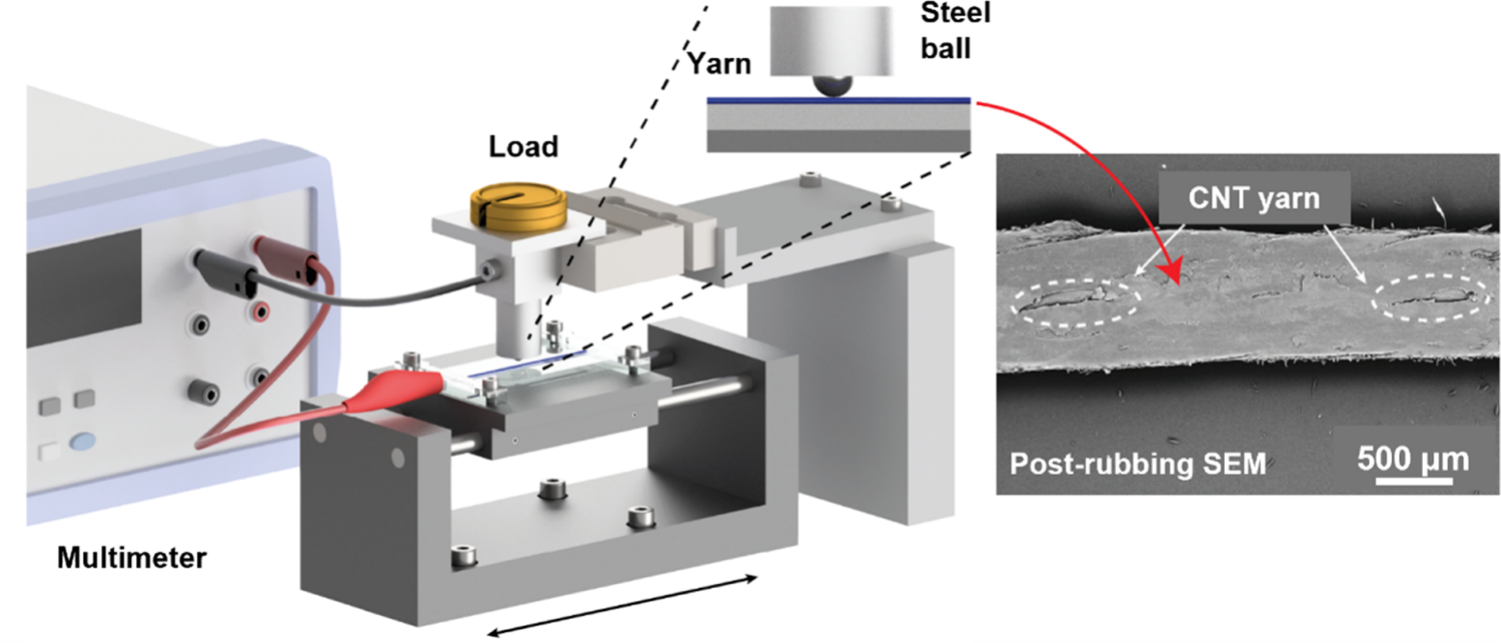
Schematic of friction measurement with an SEM image of the triboelectric yarn after the wear resistance test. The dashed circles show the exposed CNT yarn. The figure has been partially adapted from an already existing work.^6^

The wear resistance of a triboelectric yarn is rarely evaluated despite being a crucial parameter to demonstrate its suitability to the industrial manufacturing process and real-life applications. Abrasion testing of the PA11 triboelectric was conducted by repeatedly rubbing the yarn with a steel ball while measuring the electrical resistance between the CNT core and the steel ball (Figure 5). The drop in resistance over time was attributed to the progressive damage of the PA11-coating.

**Figure 6:**
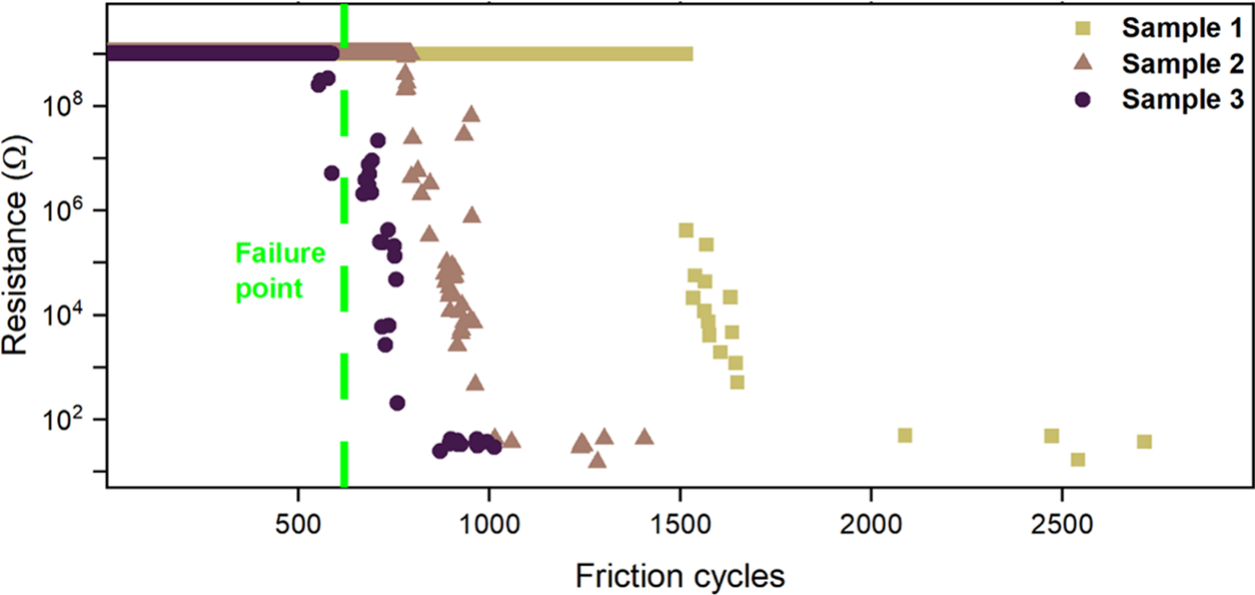
Resistance measurements between the triboelectric yarn and the steel ball were taken across friction cycles. The dashed line highlights the failure point where the coating begins to delaminate.

Figure 6 shows the electrical resistance between the CNT yarn and steel ball during the rubbing cycles. Three samples were tested, which started failing after approximately 600, 900, and 1600 cycles. The PA11 triboelectric yarn showed remarkable wear resistance, considering the applied stress of 6 MPa. The postexperiment images showed that the PA11 fibers were deformed into a film-like structure, and multiple rupture points were visible in the coating. The PA11 triboelectric yarn showed comparable wear resistance to previously reported electrospun triboelectric yarns.^6,20^ On average, the PA11 yarn failed after a lower number of cycles than the PVDF fiber-based yarns, most likely because of differences in the individual fibers as well as in the fiber network. The Young’s modulus of bulk PVDF is approximately twice that of bulk PA11 (2.4 and 1.2 GPa, respectively).^38^ Furthermore, the PVDF fiber-based yarns had a roughly 30% thicker coating and 10 times larger individual fiber diameter compared to the PA11 yarn. The high fatigue and wear resistance of the PA11 triboelectric yarn were linked to the bonding between the CNT yarn and the PA11 fibers. Raman spectroscopy was used to investigate whether chemical bonding between the CNTs and PA11 fibers had occurred. The data in Figure S6 are without any significant peak shift, displaying no evidence of chemical bonding between the CNT yarn and the PA11 fibers on the triboelectric yarn. Therefore, mechanical interlocking between the CNT yarn and PA11 fibers is the main bonding force. The results are consistent with Raman data of the previously reported PVDF triboelectric yarn.^6^

The effect of water on the PA11 triboelectric yarn was investigated by testing its power output after 5 and 10 washing cycles. Figure S7 shows that the power output of the triboelectric yarn was not affected by washing. SEM images of the yarn displayed a slight increase in the yarn diameter (4%), but no signs of coating damage. The results demonstrate that PA11 fibers did not undergo degradation *via* hydrolysis and that the washing cycles did not significantly affect the triboelectric yarn power output. The slight increase in the yarn diameter is related to repeated tapping, as seen in the fatigue tests. Exposure to water is known to damage nylons, reducing their mechanical and electrical properties.^39^ However, PA11 is more water-resistant than lower number PAs (*e.g.*, PA6 and PA7).^39^ Furthermore, δ′ phase PA11 has been reported to have higher water resistance than α phase PA11.^40^ PA11 has good resistance to alkaline solutions; hence, detergents are unlikely to damage the triboelectric yarn.^41^ The PA11 triboelectric yarn showed similar water resistance (after 10 washing cycles) as other high-performance triboelectric yarns.^42,43^

### Energy-Harvesting and Human–Computer Interface Applications

**Figure 7:**
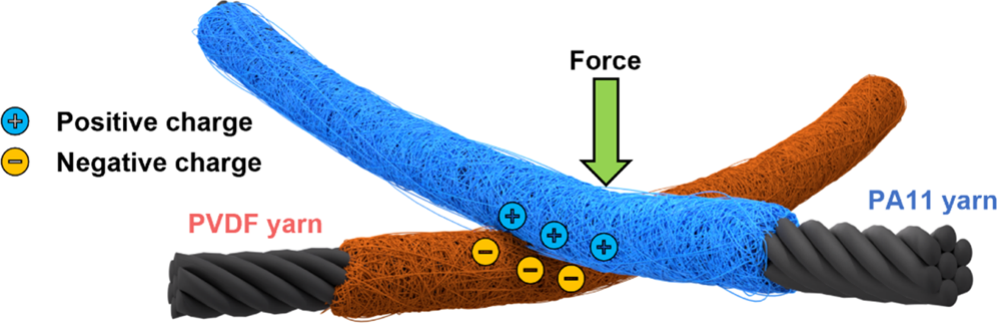
Operating principle of the triboelectric textile comprised of two core–shell yarns, tribonegative PVDF and tribopositive PA11.

Triboelectric textiles can be used as self-powered force and touch sensors and more generally in haptic devices for the human–computer interface. The working principle of a textile-based triboelectric device is shown in Figure 7.

**Figure 8:**
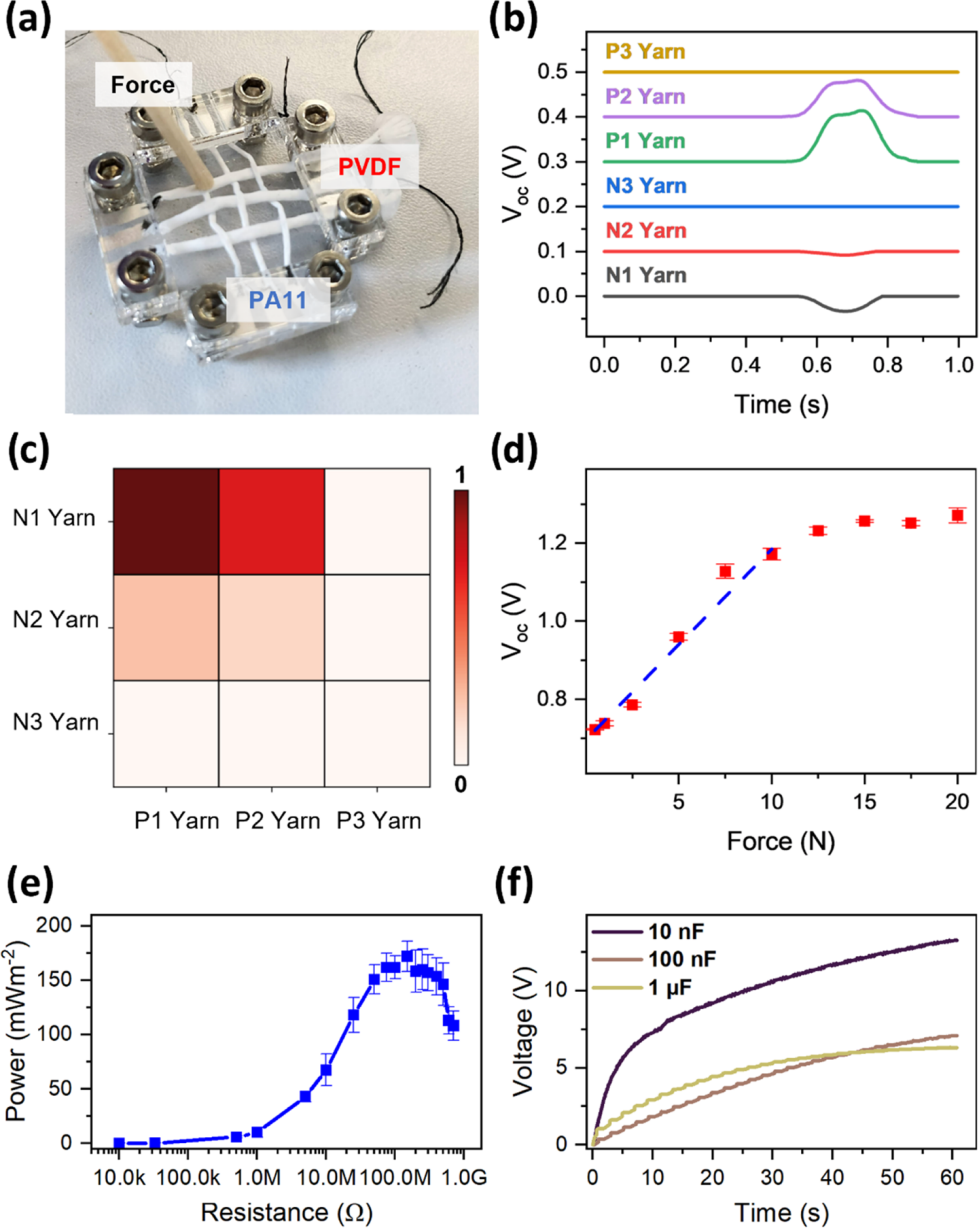
Energy-harvesting and sensing applications of a triboelectric textile. Each set of yarns (*i.e.*, PVDF and PA11) is connected in parallel and is connected to a multimeter lead. (a) Photograph of the triboelectric textile made by weaving PVDF and PA11 yarns together with touch sensing capabilities. (b) Demonstration of touch sensing capabilities: P1–3 describe PVDF yarn and N1–3 describe PA11 yarns. *V*_oc_ signal of the respective yarn during touch. The voltage of each yarn was filtered and smoothed for easier signal detection, and each yarn was configured in a single-electrode mode. (c) Spatial map of the signal of the triboelectric textile during touching. The pixel color refers to the normalized intensity of *V*_oc_ produced between the respective yarns. (d) Peak power density of the triboelectric textile. (e) Force sensing measurement of the triboelectric textile using open-circuit voltage (*V*_oc_*)*. A load cell was used to measure the applied force. The dotted line shows the linear fit from which the force and *V*_oc_ coefficient were calculated. (f) Capacitor charging curves using the triboelectric textile of three different capacitors. A full-bridge rectifier was used to rectify the output.

PA11 triboelectric yarns were woven with PVDF triboelectric yarns to create a functional triboelectric textile (Figure 8a). The PVDF yarns were prepared using procedures reported earlier. Three yarns of each kind were woven using a plain weave configuration, where the weft thread passed over the warp in an “over and under” sequence. The triboelectric textile was pressed down by using an aluminum block at 2 N and 2 Hz. During contact, the PA11 yarns were pressed against the PVDF yarns at the crossover points, as shown in Figure 8a, where a textile-based touchpad demonstrating practical use is presented. The touchpad worked by measuring the *V*_oc_ of each yarn and combining the signals to determine the position of the touch. The *V*_oc_ of the individual yarns during a single touch is shown in Figure 8b. Subsequently, it is possible to create a spatial map by multiplying together the *V*_oc_ values of different yarns (Figure 8c). The spatial map results showed a clear correlation with the location of the touch, showcasing the potential of the triboelectric textile as a haptic device. For example, the proof-of-concept triboelectric textile could be integrated into garments or upholstery to enable a more seamless and immersive interface to mixed and virtual reality. The *V*_oc_ value of the triboelectric yarn is directly related to the applied force. Therefore, the force sensing capability of the triboelectric textile was investigated (Figure 8d). The results showed a nearly linear relationship between force and *V*_oc_ until 10 N, with a coefficient of 50 ∼mVN^–1^. Beyond 10 N, the response became nonlinear and plateaued after 15 N, demonstrating the upper limit of the triboelectric textile force sensing capabilities.

The energy-harvesting properties of the triboelectric textile were characterized by measuring the power output and capacitor charging time. All yarns with the same coating (PVDF or PA11) were connected in parallel to maximize the power output. Figure 8e shows that the triboelectric textile had an estimated maximum peak power density of 172 mW m^–2^ across a 150 MΩ load resistance (contact area: 0.9 mm^2^, mechanical input: 2 N at 2 Hz). Despite being a proof-of-concept device, the triboelectric textile showed 1.8 times higher peak power density than previously reported woven electrospun yarns (∼93 mW m^–2^, mechanical input: 200 N and 3 Hz).^3^ The capacitor charging test showed that the triboelectric textile was able to charge a 1 μF capacitor to 5 V in approximately 30 s (Figure 8f).

## Conclusions

In this work, we fabricated highly reproducible PA11 fibers with exceptional mechanical properties, rich in polar γ and δ′ phases and yarns suitable for real-world applications. High mechanical stability was obtained by the regulation of RH during electrospinning. DSC results showed a highly crystalline structure that was advantageous for the material’s performance. Chemical analysis confirmed the combination of γ and δ′ phases in the fibers, which was further validated with KPFM, demonstrating the high surface potential stability of PA11. Through electrospinning, we fabricated a novel triboelectric yarn using a conductive CNT core that displayed outstanding energy-harvesting performance and durability. After 200,000 fatigue cycles, the power output of the triboelectric yarn increased by 30%, and it withstood over 600 rubbing cycles with high wear resistance. The triboelectric yarn also maintained its coating and power output after repeated washing cycles. By weaving PA11 and PVDF triboelectric yarns together, we created an exceptional and functional triboelectric textile with practical applications in the energy-harvesting and human–computer interface.

## Experimental Section

### Materials and Methods

#### Solution Preparation

A 10 wt % solution was prepared using PA11 pellets (Sigma-Aldrich, Germany), which were dissolved in a 60:40 mol % solution of TFA (Sigma-Aldrich, Germany) and acetone (analytical standard, Avantor, Poland). The solution was heated to 40 °C and stirred for 3 h at a constant speed of 300 rpm using a hot plate (IKA RCT basic, Germany). The CNT yarn was purchased from DexMat Inc. and was used as received.

### Electrospinning

To fabricate the triboelectric yarn, electrospinning was conducted using an EC-DIG electrospinner with a climate control system (IME, Netherlands). The collector was modified to accommodate the CNT yarn. The setup description can be found in a previous work.^6^ During the electrospinning process, a stainless steel needle located 90 mm away from the grounded CNT yarn was charged with +15 kV. The solution was applied at a flow rate of 0.05 mL min^–1^ for all samples, and the electrospinning time was 30 min. The electrospinning environment was maintained at *T* = 21 °C and RH = 50%. The CNT rotation speed was set at 500 rpm. For testing of just PA11 fibers, electrospinning was carried out on a collector with an Al foil using the same settings as for the yarns.

### Film Fabrication

PA11 films were prepared as references containing γ and δ’ phases following previously reported protocols.^25^ The γ phase PA11 film was prepared by dissolving 10 wt % of PA11 in TFA. The solution was heated to 40 °C and stirred at 300 rpm for 3 h. The film was fabricated by drop-casting 0.1 mL of the solution on a silicon wafer, followed by air-drying overnight. The δ’ phase PA11 film was fabricated by placing PA11 pellets onto an Al foil and heating them to 230 °C using a hot plate. Upon melting, a 200 N force was applied to the film for 1 min using weights. Finally, the PA11 film was quenched in an ice bath, and the aluminum foil was peeled off.

### Microscopy

SEMs of F30, F50, and F70 meshes together with the triboelectric yarn cross-sectional investigation were performed using a Zeiss Merlin Gemini II SEM (Zeiss, Germany) with an accelerating voltage of 3 kV and 150 pA current at a working distance of 3–8 mm. For the cross-sectional investigation, the sample was freeze-fractured in liquid nitrogen. Before imaging, the samples were sputter-coated (Q150RS, Quorum Technologies) with a Au layer (∼10 nm) for imaging purposes. SEM on PA11-coated CNT yarns was conducted using a Hitachi TM3000 instrument (Hitachi, Japan) with an accelerating voltage of 15 kV and a working distance of 3 mm. The samples were imaged uncoated. The fiber diameter and porosity measurements were performed by utilizing ImageJ v1.5 software. Porosity was calculated by analyzing the SEM images. The images were converted to black and white with white representing fibers and black representing the background. The porosity value was determined by looking at the proportion of white and black pixels in each image. The same procedure was performed after the deformation of yarns to calculate the change in material porosity. To determine the thickness of the PA11 fiber mats, an optical microscope (Bruker Senterra) was used. The thickness was measured by finding the vertical distance between the aluminum foil and the top of the fiber mat. Five measurements were taken from different areas of the sample, and their average was calculated.

### Differential Scanning Calorimetry

Differential scanning calorimetry (DSC) measurements were carried out using a TA QA2000 DSC (TA Instruments) instrument with samples sealed in Al crucibles. The heating temperature was 5 °C min^–1^. The crystallinity value was calculated by integrating the area under the melting peak after baseline subtraction using the TA Universal Analysis software. The reference value of enthalpy of fusion of 100% crystalline polymer was 189 J g^–1^.^44^ Crystallinity was calculated following an already existing protocol used by Zhang et al.^44^

### X-ray Diffraction

Wide-angle X-ray diffraction (XRD) was carried out using a Bruker D8 (Bruker) diffractometer in Bragg–Brentano geometry with a Cu Kα X-ray source (λ = 1.5406 Å). The PA11 fibers were electrospun on a 15 mm × 15 mm Si wafer. The (400) Si reflection (32.96°) was used to calibrate the peak position, as variations in the sample height cause a peak shift. XRD data were normalized, and peak analysis was performed using Python and OriginPro (version 9.7.0.188, OriginLab Corporation) software.

### Spectroscopy

Raman spectroscopy was carried out using a 785 nm laser and a Bruker Senterra Raman microscope (Bruker). The scattered light was coupled into a ×20-long working distance objective, and a 1200 lines/mm grating was used. The laser power and acquisition time were varied between CNT yarn, triboelectric yarn, and PA11 mats to avoid sample damage. FTIR spectra were obtained using a Bruker Tensor 27 IR spectrometer (Bruker), equipped with an attenuated total internal reflection attachment (ATR).

### Mechanical Properties

Tensile testing of the PA11 fibers was performed to evaluate their mechanical properties. The samples were prepared by electrospinning the fibers directly onto rectangular paper frames (20 mm × 8 mm). The frames were subsequently mounted on a tensile machine (Kammrath and Weiss, Germany). The tensile tests were conducted at room temperature using a 1 N load cell with an extension rate of 25 μm s^–1^. The stress curve was calculated by dividing the recorded force by the cross-sectional area of the electrospun fibers mat. The cross-sectional area was estimated by measuring the mat’s thickness with a light microscope (Axio Imager, Zeiss, Germany). Data analysis was performed using OriginPro (v9.7.0.188, OriginLab Corporation) software. Figure S8 presents the raw stress–strain curves used for calculations.

### Energy-Harvesting Characterization

The energy-harvesting performance of triboelectric devices was measured by using a bespoke energy-harvesting measurement setup. The output voltage and current were recorded *via* a multimeter (Keithley 2002, Keithley Instruments) and a picoammeter (Keithley 6485, Keithley Instruments), respectively. The PA11 fiber-based devices were cyclically tapped against an aluminum block. The aluminum foil used as a collector during electrospinning supported the fibers. The PA11 yarn-based devices were cyclically tapped against a PTFE film by using a linear motor (LinMot). The PTFE film (Goodfellow) was a circular disc, 12 mm in diameter and 100 μm in thickness, and it was sputter-coated with gold on its noncontact side to create a vertical-separation triboelectric generator. The triboelectric yarn and triboelectric textile were supported by using a poly(methyl methacrylate) holder. The tapping parameters were 2 and 10 N at 2 Hz for PA11 fibers and yarn device, respectively. The measurements were recorded after 30 min of tapping in order to stabilize the electrical output. All energy-harvesting results are the average of at least three samples measured across different days. The contact area was estimated by analyzing the SEM images using ImageJ software. The images were manually cropped to the region containing fibers and then divided within the grayscale range. White pixels represented the fibers, and black pixels represented the background/empty space. Anomalous features not successfully eliminated by automated background removal were manually deleted before area measurement. The contact area was determined from the area and area fraction of the white to black pixels of each micrograph.

### Atomic Force Microscopy

We conducted atomic force microscopy (AFM), Kelvin probe force microscopy measurements using CoreAFM (Nanosurf, Switzerland) equipment. For KPFM, we used conductive HQ:NSC18/Pt (MikroMasch, Bulgaria) tips with a spring constant of *k* = 2.8 N m^–1^ and a resonance frequency of 75 kHz. During the KPFM measurements, topography data were obtained simultaneously. KPFM data are the average of scan lines taken on the uppermost section of 14 different fibers, which were measured in five separate scan regions. To serve as a control measurement of the KPFM signal, we measured the ITO substrate at the same time. The surface potential for ITO remained stable for all samples with a standard deviation of only 4 mV. All measurements were conducted in a single session to ensure similar room conditions. The room conditions during testing were RH = 20–30% and T = 22 °C. An average of five lines for each region was taken to obtain the ITO surface potential values. To ensure reliable results, only data from the apex of individual fibers were used for calculations of KPFM. All AFM data were processed by using Gwyddion (v2.56, gwyddion.net) and OriginPro software.

### Wear Resistance Characterization

The triboelectric yarn wear resistance was measured by using a custom setup (see Figure 5). First, the yarn was fixed using clamps, and the entire assembly was attached to a linear motor. Next, the rubbing was performed using a hardened chrome steel ball (5 mm diameter) attached to the static assembly. Finally, the yarn and the steel ball were connected to a multimeter (Keithley 2002, Keithley Instruments) to measure the electrical resistance between them. The steel ball contact area was approximately 1.6 cm^2^, and the applied force on the yarn was 1215 N.

### Water Resistance Characterization

Water resistance characterization was performed by washing the triboelectric yarn in a beaker full of water at 30 °C. The solution was stirred at 400 rpm using a magnetic stirrer (IKA RCT basic, Germany). Each washing cycle comprised a 10 min washing period followed by air-drying.

## Data Availability

Data related to this study, including raw data and analysis files, are available from the corresponding author upon reasonable request.

## Supporting Information Available

SEM images and diameter distribution of electrospun PA11 fibers; FTIR results for structural characterization of PA11; AFM and KPFM scan of a PA11 fiber; SEM of PA11 fibers electrospun on CNT yarn with diameter distribution; schematic representation illustrating the differences in power measurements; Raman spectroscopy spectra; power output of PA11 triboelectric yarn after washing; stress-strain plots of tensile tested PA11 fiber networks; mechanical properties of Nylon-11 as calculated from tensile testing data (PDF)
