# Supplementary material for: Wear-Resistant Smart Textiles Using Nylon-11 Triboelectric Yarns
Source: ACS Appl Mater Interfaces. 2023 Nov 20;15(48):56575–86. doi: 10.1021/acsami.3c14156 (PMC10711711; doi:10.1021/acsami.3c14156)
Supplement: Supplementary file 1 — am3c14156_si_001.pdf [file am3c14156_si_001.pdf]

Supporting information

Wear-Resistant Smart Textiles Using Nylon-11 Triboelectric Yarns

Piotr K. Szewczyk[1]†, Tommaso Busolo[2]†, Sohini Kar-Narayan[2], Urszula Stachewicz[1]*

1 Faculty of Metals Engineering and Industrial Computer Science, AGH University of Krakow, Krakow 30-059, Poland

2 Department of Materials Science & Metallurgy, University of Cambridge, Cambridge CB3 0FS, United Kingdom

† Piotr K. Szewczyk and Tommaso Busolo contributed equally to the manuscript

* Corresponding author email: ustachew@agh.edu.pl

Additional experimental results and supporting table and figures:

Figure S1. SEM images and diameter distribution of electrospun PA11 fibers.

Figure S2. FTIR results for structural characterization of PA11.

Figure S3. AFM and KPFM scan of a PA11 fiber.

Figure S4. SEM of PA11 fibers electrospun on CNT yarn with diameter distribution.

Figure S5. A schematic representation illustrating the differences in power measurements.

Figure S6. Raman spectroscopy spectra.

Figure S7. Power output of PA11 triboelectric yarn after washing.

Figure S8. Stress-strain plots of tensile tested PA11 fiber networks.

Table S1. Mechanical properties of Nylon-11 as calculated from tensile testing data.

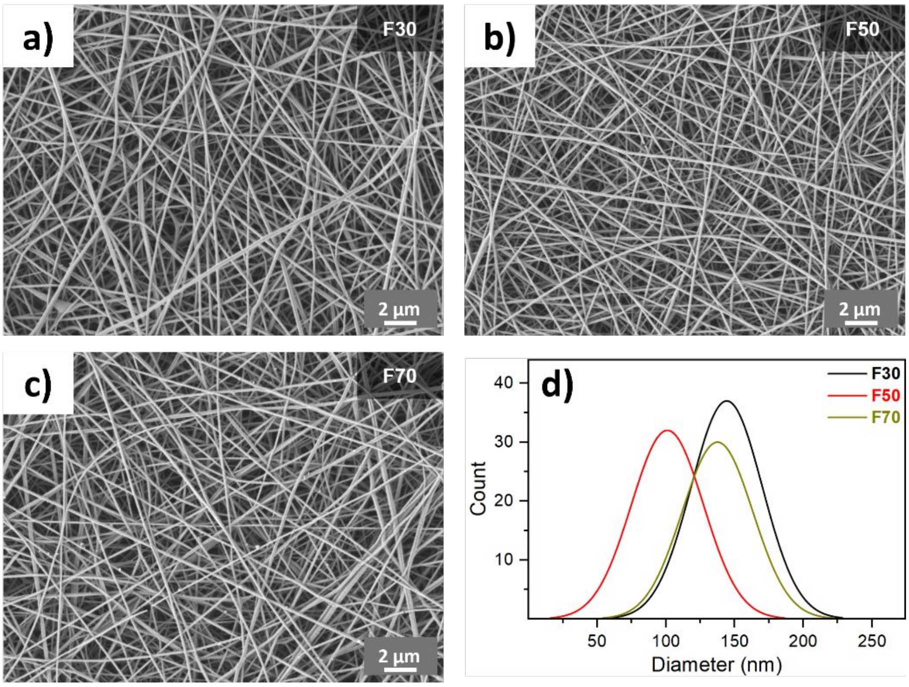

Figure S1. SEM micrographs of electrospun PA 11 samples a) F30, b) F50 c) F70 and d) diameter distribution of electrospun PA11 fibers.

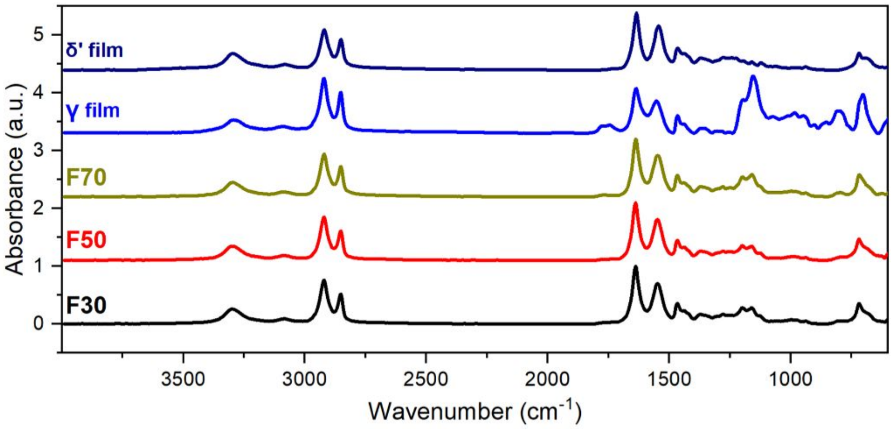

Figure S2. FTIR results for structural characterization of PA11 fibers together with γ and δ' films used for reference.

Table S1. Mechanical properties of Nylon-11 fibers calculated from tensile testing data.

	F30	F50	F70
**Ultimate tensile stress (MPa)**	1.79 ± 0.32	1.02 ± 0.14	0.97 ± 0.03
**Toughness (MPa)**	28.3 ± 6.7	25.8 ± 0.9	18.0 ± 0.1
**Strain at failure (%)**	21.2 ± 2.5	35.5 ± 1.0	28.2 ± 1.9

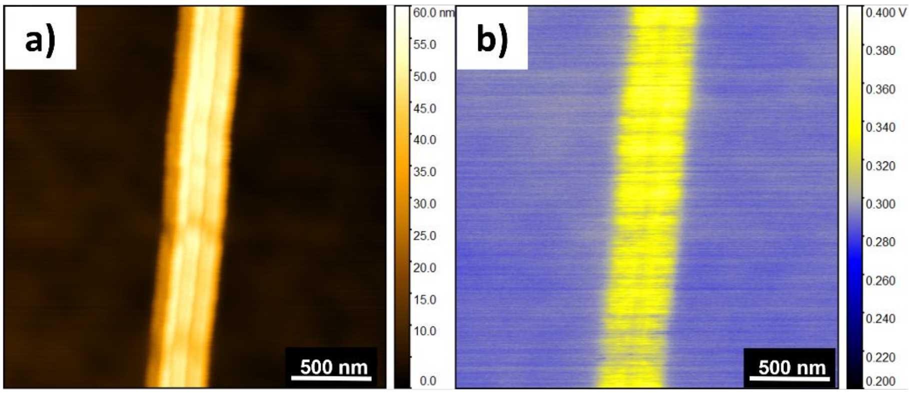

Figure S3. Exemplary a) topography and b) KPFM scans of a PA11 fiber.

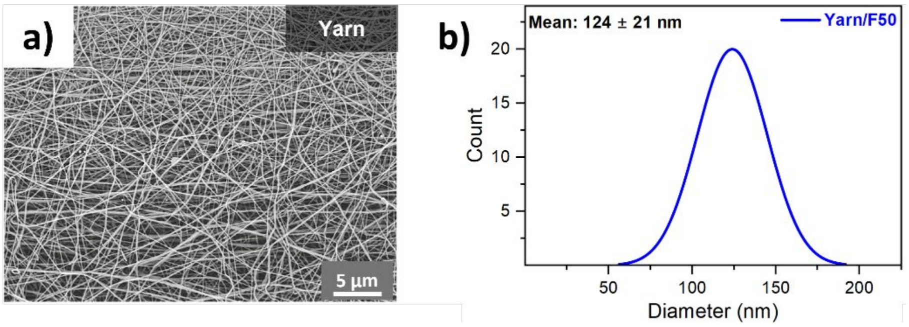

Figure S4. a) SEM of PA11 fibers electrospun on CNT yarn with b) diameter distribution.

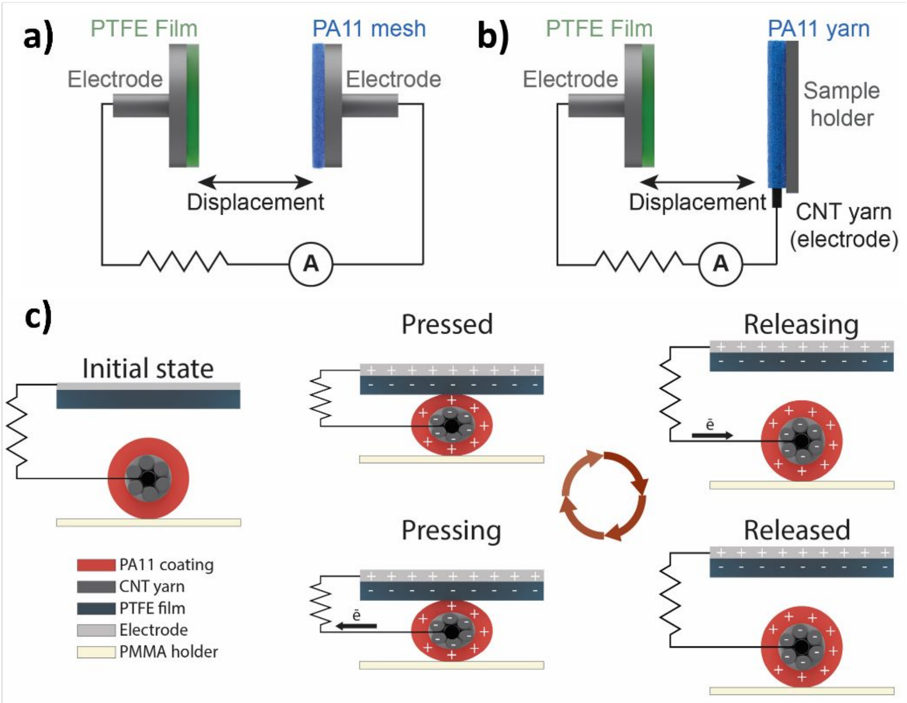

Figure S5. A schematic representation illustrating the differences in power measurements between a) mesh and b) CNT yarn with c) working principles of pressing and releasing of the PA11 triboelectric yarn.

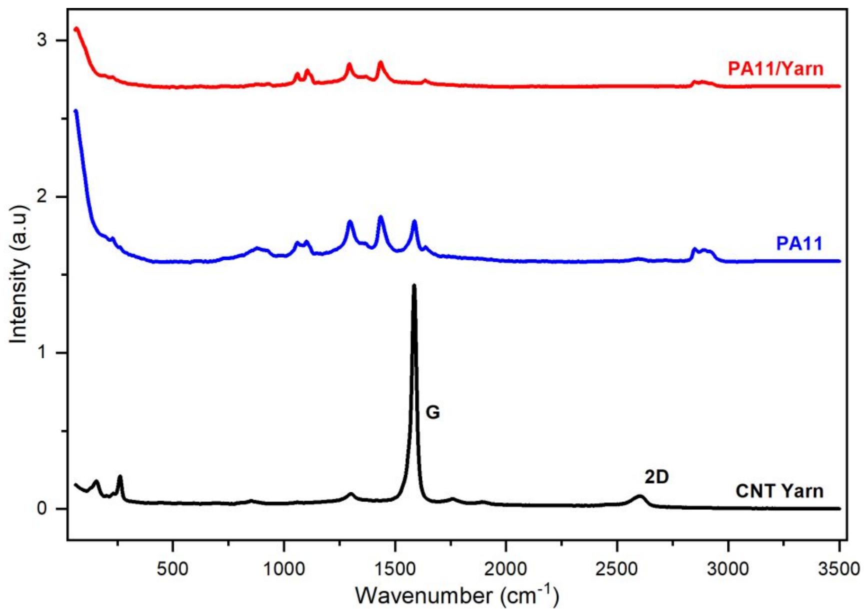

Figure S6. Raman spectroscopy spectra from the PA-11 Yarn, CNT Yarn and PA-11.

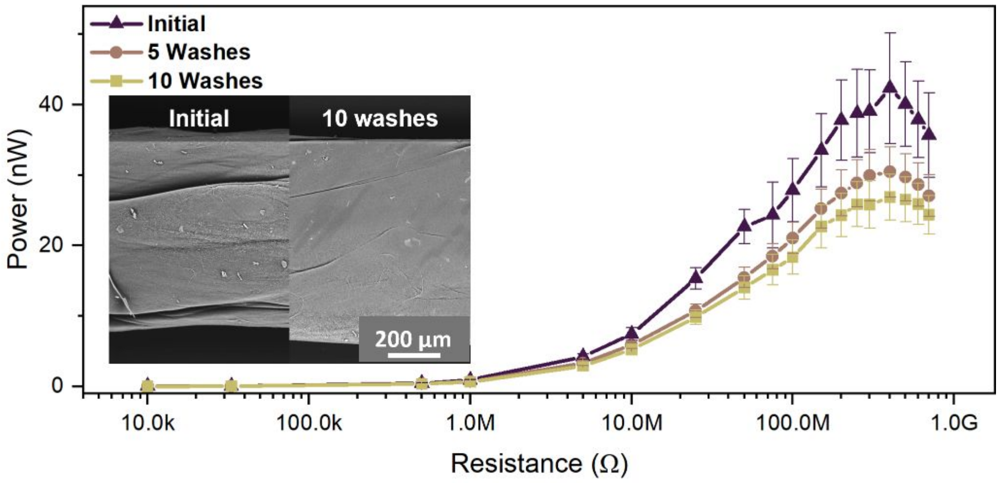

Figure S7. Power output of PA11 triboelectric yarn after washing.

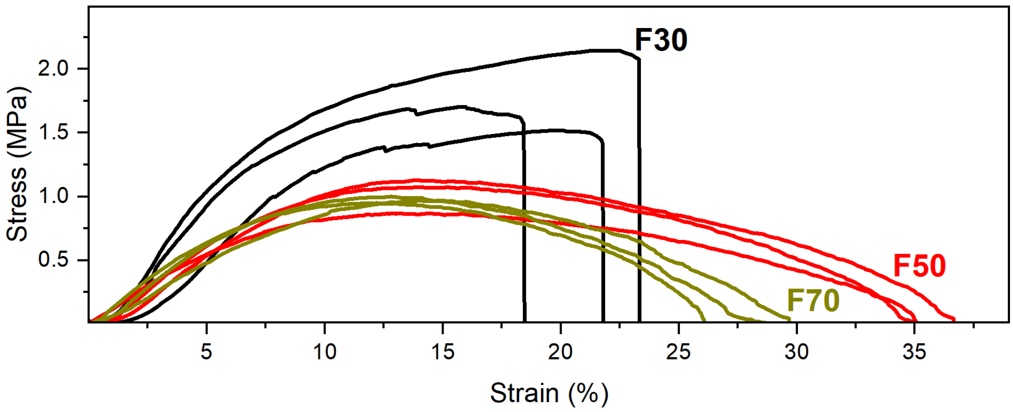

Figure S8. Stress-strain plots of tensile tested PA11 fiber networks.
